# Supplementary material for: Colour polymorphism associated with a gene duplication in male wood tiger moths
Source: eLife. 2023 Oct 30;12:e80116. doi: 10.7554/eLife.80116 (PMC10635649; doi:10.7554/eLife.80116)
Supplement: Supplementary file 1. — (A) 21 genes are within in the QTL interval. Start and end positions shown are on scaffold 206 in the yellow A. plantaginis reference genome. Gene sequences were blasted against Heliconius melpomene and searched for in FlyBase. Apla gene names from annotations produced by Yen et al., 2020. (B) Primers used for genotyping. Tested using GoTaq Flexi buffer and GoTaq DNA polymerase, with annealing temperature of 57°C for 35 cycles. ‘Ye12’ primers surround a small deletion in white alleles, producing a 163 bp product from Y alleles and 128 bp product from W alleles. ‘Dup5’ primers amplify a 449 bp sequence within the duplicated sequence only in moths with at least one W allele. See Figure 2—figure supplement 4 for gel images. (C) List of differentially expressed genes found in the linkage group containing scaffold 419 (WW reference). (D) Details of the number of eggs injected with each sgRNA and those which produced adult moths. (E) Sample list of all lab cross individuals used in linkage mapping. (F) Sample list of all wild samples used. (G) CRISPR sgRNAs sequences. (H) Elution gradient used in pheomelanin HPLC analysis. (I). Waveform of disposable working electrode in pheomelanin analysis. [file elife-80116-supp1.docx]

**Supplementary File 1**

Supplementary file 1A: 21 genes are within in the QTL interval. Start and end positions shown are on scaffold 206 in the yellow *A. plantaginis* reference genome. Gene sequences were blasted against *Heliconius melpomene* and searched for in Flybase. Apla gene names from annotations produced by Yen et al. (2020).

| **Apla gene name (yellow ref)** | **Hmel gene hit** | **% identical** | **Hmel chr** | **Dmel gene hit** | **Blast score** | **E value** |
| --- | --- | --- | --- | --- | --- | --- |
| jg6722 | HMEL032761g1.t1 | 45.8 | 15 | kis-PF | [29.261](http://flybase.org/blast/checkJobStatus.html?jobHash=6e143a76e760765cbfdf28a82b334ae1#gnl\|dmel\|FBpp0300575) | 3.32994 |
| jg6723 | unknown |  |  |  |  |  |
| jg6724 | unknown |  |  |  |  |  |
| jg6725 | HMEL032764g1.t1 | 80.9 | 15 | CG9853-PC | [234.958](http://flybase.org/blast/checkJobStatus.html?jobHash=a9ff65e4dab5266fff007104742a99d2#gnl\|dmel\|FBpp0310621) | 7.86E-62 |
| jg6726 | HMEL004965g1.t1 | 68.7 | 15 | CG8128-PB | [240.736](http://flybase.org/blast/checkJobStatus.html?jobHash=7c030f2178249064278f86d68804bdfa#gnl\|dmel\|FBpp0309990) | 9.40E-64 |
| jg6727 | HMEL004965g1.t1 | 61.2 | 15 | CG8128-PB | [240.736](http://flybase.org/blast/checkJobStatus.html?jobHash=7c030f2178249064278f86d68804bdfa#gnl\|dmel\|FBpp0309990) | 9.40E-64 |
| jg6728 | HMEL004964g1.t1 | 43.6 | 15 | CG6262-PA | [29.261](http://flybase.org/blast/checkJobStatus.html?jobHash=297770370a6d936d91a2aaf63bc17606#gnl\|dmel\|FBpp0086296) | 0.764596 |
| jg6729 | unknown |  |  |  |  |  |
| jg6730 | unknown |  |  |  |  |  |
| jg6731 | HMEL004962g1.t1 | 81.4 | 15 | dimm-PB | [105.145](http://flybase.org/blast/checkJobStatus.html?jobHash=f89705351f38b6a23de2e107c120dd26#gnl\|dmel\|FBpp0305546) | 4.56E-23 |
| jg6732 | HMEL016957g1.t1 | 62.4 | 19 | CkIalpha-PG | [512.301](http://flybase.org/blast/checkJobStatus.html?jobHash=d8d29048cfcfa473948815130098f679#gnl\|dmel\|FBpp0311373) | 2.35E-145 |
| jg6733 | unknown |  |  |  |  |  |
| jg6734 | HMEL004960g1.t1 | 69.7 | 15 | CG7322-PC | [184.882](http://flybase.org/blast/checkJobStatus.html?jobHash=aa75bbe8d98381994144cc9e71586fa8#gnl\|dmel\|FBpp0311891) | 5.40E-47 |
| jg6735 | HMEL032767g1.t1 | 85.2 | 15 | CG7322-PC | [209.92](http://flybase.org/blast/checkJobStatus.html?jobHash=c465ef5abc34dbbbc05a669c2516ffee#gnl\|dmel\|FBpp0311891) | 1.57E-54 |
| jg6736 | HMEL010407g1.t1 | 75 | 15 | CG10311-PB | [142.51](http://flybase.org/blast/checkJobStatus.html?jobHash=58f088ab99dcbb94f5c447461033df94#gnl\|dmel\|FBpp0311397) | 1.76E-34 |
| jg6737 | HMEL032770g1.t1 | 59.7 | 15 | CG34307-PB | [39.2762](http://flybase.org/blast/checkJobStatus.html?jobHash=3e2f188daef4070c8410f478aecee7b1#gnl\|dmel\|FBpp0290301) | 0.00975671 |
| jg6738 | HMEL010409g1.t1 | 72.1 | 15 | yellow h |  |  |
| jg6739 | HMEL010409g1.t1 | 46 | 15 | yellow h | [112.464](http://flybase.org/blast/checkJobStatus.html?jobHash=46399e568c8870693705ddd2e06234c3#gnl\|dmel\|FBpp0088184) | 1.74E-25 |
| jg6740 | HMEL032771g1.t1 | 76 | 15 | CG8401-PB | [35.8094](http://flybase.org/blast/checkJobStatus.html?jobHash=a96cc187afd39a50184f3c9c5e34d281#gnl\|dmel\|FBpp0290414) | 0.0810412 |
| jg6741 | HMEL002092g1.t1 | 68.4 | 15 | yellow d2 | [290.426](http://flybase.org/blast/checkJobStatus.html?jobHash=e94fe84f418e393c4f308641f176adeb#gnl\|dmel\|FBpp0071921) | 2.27E-78 |
| jg6742 | HMEL032773g1.t1 | 86.7 | 15 | yellow e | [387.882](http://flybase.org/blast/checkJobStatus.html?jobHash=48e62e2987412b3d161e5b97bf1e273b#gnl\|dmel\|FBpp0082225) | 8.79E-108 |

Supplementary file 1B: Primers used for genotyping. Tested using GoTaq Flexi buffer and GoTaq DNA polymerase, with annealing temperature of 57^o^C for 35 cycles. ‘Ye12’ primers surround a small deletion in white alleles, producing a 163bp product from Y alleles and 128bp product from W alleles. ‘Dup5’ primers amplify a 449bp sequence within the duplicated sequence only in moths with at least one W allele. See Appendix figure 1 for gel images.

| **Primer name** | **Primer sequence** | **Position in YY genome** | **Position in WW genome** |
| --- | --- | --- | --- |
| ye12_F | ATGGCCGATTACGTCTTACGAC | YY_tarseq_206_arrow:9846212 | WW_tarseq_419_arrow:7150189 |
| ye12_R | CAACTAAATACAAAGTAGCTTCCCT | YY_tarseq_206_arrow:9846375 | WW_tarseq_419_arrow:7150353 |
| dup5C_f | ACTGACGTTTGTTTTGTCCCAA | NA | WW_tarseq_419_arrow:7052355 |
| dup5D_R | GGTGTGCATATTCCTGCTGT | NA | WW_tarseq_419_arrow:7052785 |

Supplementary file 1C: List of differentially expressed genes found in the linkage group containing scaffold 419 (WW reference).

| ***A. pla. genes*** | ***Scaffold*** | ***Orthogroup*** | ***D. mel. gene id*** | ***D. mel. gene name*** | ***logFC*** | ***adj.P.Val*** | ***Stage*** |
| --- | --- | --- | --- | --- | --- | --- | --- |
| *jg14802* | WW_tarseq_540_arrow | OG0000503 | NP_651812.1, NP_001263113.1, NP_001189282.1 | epidermal stripes and patches, isoform B [Drosophila melanogaster] | 3.04 | 0.04 | premel |
| *jg15101* | WW_tarseq_540_arrow | OG0000975 | NP_001260005.1 | uncharacterized protein Dmel_CG43707, isoform E [Drosophila melanogaster] | -3.88 | 0 | mel |
| *jg1308* | WW_tarseq_419_arrow | OG0001450 | NP_524344.1 | yellow-e | 10.31 | 0.01 | premel |
| *jg1310* | WW_tarseq_419_arrow | OG0001450 | NP_524344.1 | yellow-e | 3.86 | 0.02 | premel |
| *jg2035* | WW_tarseq_531_arrow | OG0007293 | NP_732407.1 | cryptochrome [Drosophila melanogaster] | 2.95 | 0.05 | premel |
| *jg15103* | WW_tarseq_540_arrow | OG0007341 | NP_609535.1 | WD repeat domain 81 [Drosophila melanogaster] | 1.64 | 0.02 | 72h |
| *jg15168* | WW_tarseq_540_arrow | OG0007353 | NP_572341.1 | uncharacterized protein Dmel_CG3184 [Drosophila melanogaster] | 2.21 | 0.03 | 5days |
| *jg1153* | WW_tarseq_419_arrow |  |  |  | -6.95 | 0 | mel |
| *jg14032* | WW_tarseq_487_arrow |  |  |  | -1.7 | 0.04 | premel |
| *jg2034* | WW_tarseq_531_arrow |  |  |  | 2.49 | 0.04 | premel |
| *jg8680* | WW_tarseq_472_arrow |  |  |  | 3.42 | 0 | 5days |
| *jg9028* | WW_tarseq_472_arrow |  |  |  | 3.24 | 0.04 | mel |

Supplementary file 1D: Details of the number of eggs injected with each sgRNA and those which produced adult moths.

| **sgRNAs** | **Number_eggs_injected** | **Number_larvae** | **Percentage_hatched** | **Number_pupae** | **Number_adults** | **Hatched_to_adult**  **percentage** | **Percentage of emerged adults with phenotype** |
| --- | --- | --- | --- | --- | --- | --- | --- |
| Val1A | 109 | 29 | 26.61 | 3 | 2 | 6.9 | 100% inc. pupa |
| Val2A | 58 | 21 | 36.21 | 0 | 0 | 0.0 |  |
| Val2B | 112 | 8 | 7.14 | 1 | 1 | 12.5 | 100% (mosaic female) |
| Val3A | 216 | 11 | 5.09 | 2 | 2 | 18.2 | 50% |
| Val3B | 309 | 20 | 6.47 | 2 | 1 | 5.0 | 100% inc. pupa |
| Val1A + Val2A | 65 | 4 | 6.15 | 0 | 0 | 0.0 |  |
| Val1A + Val2B | 21 | 6 | 28.57 | 0 | 0 | 0.0 |  |
| Val1A + Val3A | 61 | 5 | 8.20 | 0 | 0 | 0.0 |  |
| Val2A + Val2B | 55 | 9 | 16.36 | 0 | 0 | 0.0 |  |
| Val2B + Val3A | 49 | 6 | 12.24 | 0 | 0 | 0.0 |  |
| Val3A + Val2B | 53 | 17 | 32.08 | 0 | 0 | 0.0 |  |
| Val3A + Val3B | 115 | 6 | 5.22 | 0 | 0 | 0.0 |  |

Supplementary file 1E: Sample list of all lab cross individuals used in linkage mapping.

| **ID** | **Sex** | **Genotype** | **Family number** | **Generation** | **Total sequenced reads** | **YY mapped reads** | **YY % mapped reads** | **YY duplication** | **YY reads retained after deduplication** | **WW mapped reads** | **WW % mapped** | **WW duplication** | **WW reads retained after deduplication** |
| --- | --- | --- | --- | --- | --- | --- | --- | --- | --- | --- | --- | --- | --- |
| FI17_3_34_1 | m | WY | 29 | Parent | 2955970 | 2715026 | 91.849 | 0.855 | 393347 | 2748494 | 92.98 | 0.80 | 544140 |
| FI17_3_83_11 | f | YY | 29 | Parent | 3197702 | 3136607 | 98.089 | 0.841 | 499345 | 3147370 | 98.43 | 0.76 | 823864 |
| FI18_1_29_1 | m | YY | 29 | F1 | 1999390 | 1946873 | 97.373 | 0.824 | 342770 | 1971086 | 98.58 | 0.83 | 342138 |
| FI18_1_29_101 | m | YY | 29 | F1 | 2361064 | 2248858 | 95.248 | 0.820 | 404408 | 2277871 | 96.48 | 0.82 | 404261 |
| FI18_1_29_11 | m | YY | 29 | F1 | 2068918 | 2015804 | 97.433 | 0.827 | 348902 | 2042671 | 98.73 | 0.83 | 348176 |
| FI18_1_29_19 | m | WY | 29 | F1 | 1146544 | 1115347 | 97.279 | 0.744 | 285992 | 1130950 | 98.64 | 0.75 | 285547 |
| FI18_1_29_2 | m | YY | 29 | F1 | 3529048 | 3407536 | 96.557 | 0.827 | 590114 | 3454443 | 97.89 | 0.83 | 591096 |
| FI18_1_29_23 | m | YY | 29 | F1 | 1283616 | 1242794 | 96.820 | 0.740 | 323494 | 1259702 | 98.14 | 0.74 | 323404 |
| FI18_1_29_27 | m | YY | 29 | F1 | 2399278 | 2331282 | 97.166 | 0.788 | 493892 | 2364010 | 98.53 | 0.79 | 494821 |
| FI18_1_29_32 | m | YY | 29 | F1 | 1816056 | 1767863 | 97.346 | 0.816 | 325860 | 1789547 | 98.54 | 0.82 | 324933 |
| FI18_1_29_33 | m | YY | 29 | F1 | 2164252 | 2100526 | 97.056 | 0.768 | 487796 | 2130653 | 98.45 | 0.77 | 488153 |
| FI18_1_29_38 | m | WY | 29 | F1 | 3748912 | 3647923 | 97.306 | 0.870 | 473781 | 3697017 | 98.62 | 0.87 | 471901 |
| FI18_1_29_39 | m | YY | 29 | F1 | 1695450 | 1237858 | 73.011 | 0.740 | 321648 | 1254382 | 73.99 | 0.74 | 321608 |
| FI18_1_29_4 | m | WY | 29 | F1 | 1659596 | 1617162 | 97.443 | 0.810 | 307254 | 1637651 | 98.68 | 0.81 | 306746 |
| FI18_1_29_41 | m | WY | 29 | F1 | 1067638 | 1035473 | 96.987 | 0.703 | 307780 | 1050028 | 98.35 | 0.71 | 307601 |
| FI18_1_29_43 | m | WY | 29 | F1 | 1180652 | 1130239 | 95.730 | 0.756 | 276252 | 1145620 | 97.03 | 0.76 | 275715 |
| FI18_1_29_44 | m | WY | 29 | F1 | 2891056 | 2818722 | 97.498 | 0.820 | 506972 | 2854834 | 98.75 | 0.82 | 507386 |
| FI18_1_29_46 | m | YY | 29 | F1 | 3147978 | 3067342 | 97.438 | 0.820 | 551441 | 3107076 | 98.70 | 0.82 | 552171 |
| FI18_1_29_49 | m | WY | 29 | F1 | 794124 | 770168 | 96.983 | 0.686 | 241694 | 780562 | 98.29 | 0.69 | 241493 |
| FI18_1_29_58 | m | YY | 29 | F1 | 2893010 | 1473576 | 50.936 | 0.706 | 432618 | 1496051 | 51.71 | 0.71 | 433794 |
| FI18_1_29_6 | m | YY | 29 | F1 | 3159060 | 3034452 | 96.056 | 0.814 | 564750 | 3077094 | 97.41 | 0.82 | 565680 |
| FI18_1_29_60 | m | WY | 29 | F1 | 1415616 | 1369725 | 96.758 | 0.762 | 326207 | 1388092 | 98.06 | 0.77 | 326034 |
| FI18_1_29_65 | m | YY | 29 | F1 | 3140294 | 3051117 | 97.160 | 0.841 | 484739 | 3093059 | 98.50 | 0.84 | 484175 |
| FI18_1_29_68 | m | YY | 29 | F1 | 2696202 | 2626553 | 97.417 | 0.855 | 381758 | 2657543 | 98.57 | 0.86 | 380774 |
| FI18_1_29_74 | m | YY | 29 | F1 | 1449948 | 1413194 | 97.465 | 0.777 | 314744 | 1431318 | 98.72 | 0.78 | 313807 |
| FI18_1_29_76 | m | YY | 29 | F1 | 2531304 | 2418112 | 95.528 | 0.853 | 355571 | 2447038 | 96.67 | 0.85 | 354981 |
| FI18_1_29_78 | m | YY | 29 | F1 | 1678066 | 1625019 | 96.839 | 0.779 | 359452 | 1647238 | 98.16 | 0.78 | 359548 |
| FI18_1_29_82 | m | YY | 29 | F1 | 3198008 | 3097633 | 96.861 | 0.858 | 439408 | 3136758 | 98.08 | 0.86 | 438710 |
| FI18_1_29_83 | m | YY | 29 | F1 | 3006038 | 2866236 | 95.349 | 0.830 | 486751 | 2907742 | 96.73 | 0.83 | 485152 |
| FI18_1_29_84 | m | WY | 29 | F1 | 1520480 | 1481081 | 97.409 | 0.789 | 312609 | 1499436 | 98.62 | 0.79 | 312154 |
| FI18_1_29_88 | m | YY | 29 | F1 | 2657610 | 2587124 | 97.348 | 0.822 | 460265 | 2620810 | 98.62 | 0.82 | 460215 |
| FI18_1_29_9 | m | WY | 29 | F1 | 1554302 | 1504346 | 96.786 | 0.772 | 343716 | 1525473 | 98.15 | 0.77 | 343466 |
| FI18_1_29_93 | m | WY | 29 | F1 | 1737742 | 1691286 | 97.327 | 0.810 | 321434 | 1712318 | 98.54 | 0.81 | 320585 |
| FI18_1_29_95 | m | WY | 29 | F1 | 2188534 | 2058702 | 94.068 | 0.815 | 381273 | 2085456 | 95.29 | 0.82 | 380670 |
| FI18_1_29_98 | m | YY | 29 | F1 | 2821014 | 1951884 | 69.191 | 0.755 | 478442 | 1980282 | 70.20 | 0.76 | 479244 |
| FI17_3_71_3 | m | WY | 30 | Parent | 4317516 | 4162243 | 96.404 | 0.853 | 613865 | 4224077 | 97.84 | 0.80 | 830911 |
| FI17_3_81_4 | f | YY | 30 | Parent | 3107120 | 3026320 | 97.400 | 0.857 | 433556 | 3040627 | 97.86 | 0.75 | 541186 |
| FI18_1_30_106 | m | WY | 30 | F1 | 2715010 | 2053347 | 75.629 | 0.790 | 431541 | 2083411 | 76.74 | 0.79 | 431795 |
| FI18_1_30_11 | m | WY | 30 | F1 | 2869392 | 2778003 | 96.815 | 0.848 | 423555 | 2815377 | 98.12 | 0.85 | 422981 |
| FI18_1_30_12 | m | WY | 30 | F1 | 2973814 | 2844480 | 95.651 | 0.834 | 473217 | 2886012 | 97.05 | 0.84 | 471277 |
| FI18_1_30_14 | m | WY | 30 | F1 | 4478234 | 2564695 | 57.270 | 0.832 | 431954 | 2601818 | 58.10 | 0.83 | 430023 |
| FI18_1_30_18 | m | WY | 30 | F1 | 1599220 | 1555974 | 97.296 | 0.818 | 283223 | 1577278 | 98.63 | 0.82 | 281921 |
| FI18_1_30_2 | m | YY | 30 | F1 | 3022868 | 2785048 | 92.133 | 0.823 | 492599 | 2823454 | 93.40 | 0.83 | 492254 |
| FI18_1_30_24 | m | WY | 30 | F1 | 1825228 | 1516006 | 83.058 | 0.794 | 311592 | 1538155 | 84.27 | 0.80 | 310094 |
| FI18_1_30_3 | m | YY | 30 | F1 | 1734848 | 1022616 | 58.946 | 0.809 | 195239 | 1034509 | 59.63 | 0.81 | 193181 |
| FI18_1_30_30 | m | WY | 30 | F1 | 2787488 | 2698920 | 96.823 | 0.801 | 536756 | 2740534 | 98.32 | 0.80 | 535060 |
| FI18_1_30_32 | m | YY | 30 | F1 | 2379786 | 2315421 | 97.295 | 0.827 | 400142 | 2349891 | 98.74 | 0.83 | 398925 |
| FI18_1_30_36 | m | WY | 30 | F1 | 1527360 | 1339985 | 87.732 | 0.774 | 303226 | 1359367 | 89.00 | 0.78 | 302162 |
| FI18_1_30_4 | m | WY | 30 | F1 | 2383670 | 2321238 | 97.381 | 0.836 | 381226 | 2352819 | 98.71 | 0.84 | 379064 |
| FI18_1_30_40 | m | WY | 30 | F1 | 2346222 | 2284549 | 97.371 | 0.843 | 359175 | 2315827 | 98.70 | 0.85 | 356863 |
| FI18_1_30_42 | m | YY | 30 | F1 | 1070592 | 1038205 | 96.975 | 0.768 | 241165 | 1053339 | 98.39 | 0.77 | 240238 |
| FI18_1_30_43 | m | YY | 30 | F1 | 1972550 | 1918571 | 97.263 | 0.821 | 343546 | 1946119 | 98.66 | 0.82 | 342191 |
| FI18_1_30_5 | m | WY | 30 | F1 | 1426882 | 1367090 | 95.810 | 0.788 | 290415 | 1387392 | 97.23 | 0.79 | 289031 |
| FI18_1_30_52 | m | WY | 30 | F1 | 5053696 | 4514518 | 89.331 | 0.892 | 487534 | 4574384 | 90.52 | 0.89 | 481237 |
| FI18_1_30_53 | m | YY | 30 | F1 | 2230112 | 2171645 | 97.378 | 0.818 | 396180 | 2196873 | 98.51 | 0.82 | 397275 |
| FI18_1_30_54 | m | YY | 30 | F1 | 3919162 | 3799033 | 96.935 | 0.883 | 443179 | 3851217 | 98.27 | 0.89 | 436759 |
| FI18_1_30_55 | m | WY | 30 | F1 | 2322312 | 2192761 | 94.421 | 0.818 | 399266 | 2223184 | 95.73 | 0.82 | 398852 |
| FI18_1_30_56 | m | WY | 30 | F1 | 1114538 | 1082869 | 97.159 | 0.733 | 288835 | 1098673 | 98.58 | 0.74 | 288058 |
| FI18_1_30_57 | m | WY | 30 | F1 | 3931316 | 3777836 | 96.096 | 0.868 | 500346 | 3827592 | 97.36 | 0.87 | 496429 |
| FI18_1_30_62 | m | WY | 30 | F1 | 2868180 | 2791424 | 97.324 | 0.835 | 459977 | 2830970 | 98.70 | 0.84 | 456986 |
| FI18_1_30_63 | m | YY | 30 | F1 | 1461576 | 1423021 | 97.362 | 0.790 | 299500 | 1441196 | 98.61 | 0.79 | 299667 |
| FI18_1_30_65 | m | WY | 30 | F1 | 1866314 | 1812580 | 97.121 | 0.827 | 314087 | 1838963 | 98.53 | 0.83 | 312070 |
| FI18_1_30_66 | m | WY | 30 | F1 | 3043110 | 2936318 | 96.491 | 0.896 | 305921 | 2970116 | 97.60 | 0.90 | 301052 |
| FI18_1_30_75 | m | WY | 30 | F1 | 2295070 | 2216385 | 96.572 | 0.813 | 414891 | 2249790 | 98.03 | 0.82 | 413818 |
| FI18_1_30_8 | m | WY | 30 | F1 | 3093078 | 2659531 | 85.983 | 0.789 | 562165 | 2701905 | 87.35 | 0.79 | 561879 |
| FI18_1_30_80 | m | WY | 30 | F1 | 2270474 | 2167775 | 95.477 | 0.785 | 465533 | 2201415 | 96.96 | 0.79 | 464124 |
| FI18_1_30_84 | m | WY | 30 | F1 | 3155708 | 3061936 | 97.028 | 0.832 | 515203 | 3109723 | 98.54 | 0.84 | 512912 |
| FI18_1_30_88 | m | WY | 30 | F1 | 2060846 | 1877502 | 91.103 | 0.831 | 318137 | 1904332 | 92.41 | 0.83 | 316669 |
| FI18_1_30_89 | m | WY | 30 | F1 | 4167516 | 3948027 | 94.733 | 0.859 | 557287 | 4006846 | 96.14 | 0.86 | 553226 |
| FI18_1_30_9 | m | WY | 30 | F1 | 145926 | 139007 | 95.259 | 0.468 | 73949 | 141042 | 96.65 | 0.48 | 73822 |
| FI18_1_30_90 | m | YY | 30 | F1 | 1286248 | 1252107 | 97.346 | 0.767 | 291522 | 1270035 | 98.74 | 0.77 | 291225 |
| FI18_1_30_91 | m | WY | 30 | F1 | 2326852 | 2245625 | 96.509 | 0.816 | 412438 | 2279310 | 97.96 | 0.82 | 410759 |
| FI18_1_30_92 | m | YY | 30 | F1 | 1830454 | 1770263 | 96.712 | 0.769 | 408900 | 1796330 | 98.14 | 0.77 | 408877 |
| FI18_1_30_94 | m | YY | 30 | F1 | 3836556 | 3698968 | 96.414 | 0.871 | 478601 | 3743031 | 97.56 | 0.87 | 479708 |
| FI18_1_30_95 | m | WY | 30 | F1 | 4085302 | 3956235 | 96.841 | 0.873 | 501017 | 4013996 | 98.25 | 0.88 | 495944 |
| FI17_3_97_7 | m | WY | 32 | Parent | 5704750 | 3351852 | 58.755 | 0.843 | 525582 | 3400691 | 59.61 | 0.80 | 503529 |
| FI17_3_56_2 | f | YY | 32 | Parent | 12734726 | 2216899 | 17.408 | 0.856 | 318198 | 2224846 | 17.47 | 0.80 | 445434 |
| FI18_1_32_1 | m | WY | 32 | F1 | 2713466 | 2628102 | 96.854 | 0.853 | 386373 | 2661104 | 98.07 | 0.86 | 384355 |
| FI18_1_32_10 | m | WY | 32 | F1 | 3211340 | 3131817 | 97.524 | 0.859 | 440030 | 3169547 | 98.70 | 0.86 | 437076 |
| FI18_1_32_100 | m | WY | 32 | F1 | 2651298 | 2586070 | 97.540 | 0.849 | 390910 | 2619419 | 98.80 | 0.85 | 387892 |
| FI18_1_32_11 | m | WY | 32 | F1 | 2015790 | 1953474 | 96.909 | 0.804 | 382149 | 1979883 | 98.22 | 0.81 | 380754 |
| FI18_1_32_12 | m | YY | 32 | F1 | 2420446 | 2339031 | 96.636 | 0.807 | 452120 | 2371340 | 97.97 | 0.81 | 450573 |
| FI18_1_32_17 | m | WY | 32 | F1 | 2503044 | 2419974 | 96.681 | 0.852 | 358779 | 2450754 | 97.91 | 0.85 | 356650 |
| FI18_1_32_18 | m | YY | 32 | F1 | 1672500 | 1628328 | 97.359 | 0.817 | 297256 | 1648404 | 98.56 | 0.82 | 295997 |
| FI18_1_32_23 | m | YY | 32 | F1 | 2664986 | 2484766 | 93.237 | 0.848 | 377868 | 2516373 | 94.42 | 0.85 | 375861 |
| FI18_1_32_27 | m | YY | 32 | F1 | 2594498 | 2525197 | 97.329 | 0.823 | 447059 | 2559795 | 98.66 | 0.83 | 445908 |
| FI18_1_32_30 | m | YY | 32 | F1 | 1720144 | 1675773 | 97.421 | 0.814 | 311376 | 1697540 | 98.69 | 0.82 | 310141 |
| FI18_1_32_36 | m | WY | 32 | F1 | 2220868 | 2156152 | 97.086 | 0.781 | 473166 | 2186450 | 98.45 | 0.78 | 473939 |
| FI18_1_32_38 | m | WY | 32 | F1 | 2945502 | 2866956 | 97.333 | 0.853 | 421535 | 2906212 | 98.67 | 0.86 | 418895 |
| FI18_1_32_40 | m | WY | 32 | F1 | 1945642 | 1737773 | 89.316 | 0.798 | 350668 | 1761860 | 90.55 | 0.80 | 349957 |
| FI18_1_32_44 | m | YY | 32 | F1 | 2912328 | 2813114 | 96.593 | 0.856 | 406172 | 2849472 | 97.84 | 0.86 | 402462 |
| FI18_1_32_47 | m | WY | 32 | F1 | 1805192 | 1568781 | 86.904 | 0.771 | 360027 | 1591403 | 88.16 | 0.77 | 360198 |
| FI18_1_32_50_1 | m | WY | 32 | F1 | 2343302 | 2267978 | 96.786 | 0.838 | 367425 | 2296497 | 98.00 | 0.84 | 365773 |
| FI18_1_32_51 | m | WY | 32 | F1 | 2274740 | 2089964 | 91.877 | 0.833 | 348148 | 2115979 | 93.02 | 0.84 | 346551 |
| FI18_1_32_53 | m | YY | 32 | F1 | 1364862 | 1317458 | 96.527 | 0.774 | 297759 | 1334444 | 97.77 | 0.78 | 296711 |
| FI18_1_32_56 | m | YY | 32 | F1 | 2223158 | 2018476 | 90.793 | 0.837 | 328854 | 2044373 | 91.96 | 0.84 | 326572 |
| FI18_1_32_57 | m | YY | 32 | F1 | 2584886 | 2491294 | 96.379 | 0.833 | 416906 | 2523897 | 97.64 | 0.84 | 415749 |
| FI18_1_32_58 | m | YY | 32 | F1 | 2042488 | 1987407 | 97.303 | 0.804 | 389592 | 2015607 | 98.68 | 0.81 | 388087 |
| FI18_1_32_66 | m | WY | 32 | F1 | 2861330 | 2734683 | 95.574 | 0.817 | 500515 | 2772097 | 96.88 | 0.82 | 499501 |
| FI18_1_32_67 | m | YY | 32 | F1 | 2683192 | 2573925 | 95.928 | 0.841 | 408704 | 2607463 | 97.18 | 0.84 | 406990 |
| FI18_1_32_68 | m | YY | 32 | F1 | 2192898 | 2138659 | 97.527 | 0.830 | 363067 | 2165772 | 98.76 | 0.83 | 361189 |
| FI18_1_32_70 | m | YY | 32 | F1 | 2519412 | 2446488 | 97.106 | 0.793 | 507448 | 2482049 | 98.52 | 0.80 | 507378 |
| FI18_1_32_71 | m | YY | 32 | F1 | 3308020 | 3171685 | 95.879 | 0.851 | 472947 | 3212971 | 97.13 | 0.85 | 470874 |
| FI18_1_32_78 | m | WY | 32 | F1 | 2112880 | 2062840 | 97.632 | 0.839 | 332683 | 2088674 | 98.85 | 0.84 | 330912 |
| FI18_1_32_79 | m | YY | 32 | F1 | 2518434 | 1915805 | 76.071 | 0.812 | 360674 | 1942127 | 77.12 | 0.81 | 359605 |
| FI18_1_32_80 | m | WY | 32 | F1 | 1477900 | 1224353 | 82.844 | 0.771 | 280331 | 1241392 | 84.00 | 0.77 | 279709 |
| FI18_1_32_83 | m | WY | 32 | F1 | 1117270 | 1078079 | 96.492 | 0.731 | 289881 | 1093619 | 97.88 | 0.73 | 290086 |
| FI18_1_32_88 | m | YY | 32 | F1 | 2058940 | 2004230 | 97.343 | 0.826 | 347869 | 2029105 | 98.55 | 0.83 | 346436 |
| FI18_1_32_89 | m | WY | 32 | F1 | 2449154 | 2384743 | 97.370 | 0.824 | 420528 | 2414835 | 98.60 | 0.83 | 418755 |
| FI18_1_32_9 | m | YY | 32 | F1 | 2106886 | 2039571 | 96.805 | 0.835 | 336647 | 2064595 | 97.99 | 0.84 | 334520 |
| FI17_3_72_7 | m | WY | 35 | Parent | 6985806 | 6733807 | 96.393 | 0.918 | 555217 | 6820593 | 97.64 | 0.89 | 781200 |
| FI17_3_49_2 | f | YY | 35 | Parent | 4170902 | 3996659 | 95.822 | 0.881 | 474906 | 4008431 | 96.10 | 0.84 | 653585 |
| FI18_1_35_105 | m | YY | 35 | F1 | 4022634 | 3834980 | 95.335 | 0.843 | 602628 | 3889267 | 96.68 | 0.85 | 600085 |
| FI18_1_35_106 | m | YY | 35 | F1 | 3614566 | 3030045 | 83.829 | 0.834 | 502926 | 3071448 | 84.97 | 0.84 | 501637 |
| FI18_1_35_107 | m | YY | 35 | F1 | 3346384 | 3254695 | 97.260 | 0.861 | 451300 | 3297645 | 98.54 | 0.86 | 448886 |
| FI18_1_35_108 | m | WY | 35 | F1 | 2368662 | 2285847 | 96.504 | 0.827 | 394503 | 2316049 | 97.78 | 0.83 | 393746 |
| FI18_1_35_109 | m | YY | 35 | F1 | 4079696 | 3966821 | 97.233 | 0.853 | 581428 | 4020088 | 98.54 | 0.86 | 578209 |
| FI18_1_35_11 | m | WY | 35 | F1 | 2111094 | 2000420 | 94.758 | 0.826 | 414635 | 2026564 | 96.00 | 0.83 | 352689 |
| FI18_1_35_110 | m | WY | 35 | F1 | 2458458 | 2389520 | 97.196 | 0.826 | 386197 | 2421034 | 98.48 | 0.83 | 414091 |
| FI18_1_35_111 | m | WY | 35 | F1 | 2303368 | 2222441 | 96.487 | 0.823 | 354631 | 2252832 | 97.81 | 0.83 | 384555 |
| FI18_1_35_112 | m | WY | 35 | F1 | 3711582 | 3584681 | 96.581 | 0.866 | 479803 | 3630766 | 97.82 | 0.87 | 476672 |
| FI18_1_35_113 | m | WY | 35 | F1 | 3159130 | 3069909 | 97.176 | 0.829 | 524186 | 3113018 | 98.54 | 0.83 | 522785 |
| FI18_1_35_114 | m | WY | 35 | F1 | 2286442 | 2219116 | 97.055 | 0.796 | 451671 | 2251683 | 98.48 | 0.80 | 451709 |
| FI18_1_35_115 | m | YY | 35 | F1 | 4042534 | 3556216 | 87.970 | 0.908 | 328073 | 3596944 | 88.98 | 0.91 | 323326 |
| FI18_1_35_117 | m | YY | 35 | F1 | 5095106 | 4910918 | 96.385 | 0.877 | 605879 | 4976003 | 97.66 | 0.88 | 607205 |
| FI18_1_35_118 | m | WY | 35 | F1 | 2803240 | 2713436 | 96.796 | 0.837 | 441079 | 2749086 | 98.07 | 0.84 | 439911 |
| FI18_1_35_12 | m | YY | 35 | F1 | 5122120 | 4318359 | 84.308 | 0.892 | 465882 | 4367086 | 85.26 | 0.89 | 460939 |
| FI18_1_35_124 | m | YY | 35 | F1 | 4084550 | 3981723 | 97.483 | 0.893 | 425219 | 4031405 | 98.70 | 0.90 | 419245 |
| FI18_1_35_129 | m | WY | 35 | F1 | 1868866 | 1812576 | 96.988 | 0.823 | 320517 | 1837315 | 98.31 | 0.83 | 318853 |
| FI18_1_35_130 | m | WY | 35 | F1 | 2278784 | 2220546 | 97.444 | 0.851 | 330748 | 2249967 | 98.74 | 0.85 | 328178 |
| FI18_1_35_131 | m | YY | 35 | F1 | 2508666 | 2438390 | 97.199 | 0.839 | 391789 | 2469826 | 98.45 | 0.84 | 390368 |
| FI18_1_35_14 | m | YY | 35 | F1 | 5116298 | 4944259 | 96.637 | 0.859 | 697050 | 5013442 | 97.99 | 0.86 | 694775 |
| FI18_1_35_15 | m | YY | 35 | F1 | 3019894 | 2890565 | 95.717 | 0.785 | 621455 | 2933036 | 97.12 | 0.79 | 622407 |
| FI18_1_35_16 | m | WY | 35 | F1 | 2456980 | 2387996 | 97.192 | 0.833 | 397605 | 2421394 | 98.55 | 0.84 | 395404 |
| FI18_1_35_18 | m | YY | 35 | F1 | 3912406 | 3805044 | 97.256 | 0.885 | 436710 | 3854079 | 98.51 | 0.89 | 434097 |
| FI18_1_35_201 | m | YY | 35 | F1 | 3533726 | 3429725 | 97.057 | 0.862 | 472487 | 3475867 | 98.36 | 0.86 | 470227 |
| FI18_1_35_202 | m | YY | 35 | F1 | 1548800 | 1497770 | 96.705 | 0.789 | 315985 | 1519757 | 98.12 | 0.79 | 315829 |
| FI18_1_35_203 | m | WY | 35 | F1 | 2529636 | 2361197 | 93.341 | 0.815 | 435746 | 2394939 | 94.68 | 0.82 | 434933 |
| FI18_1_35_204 | m | WY | 35 | F1 | 1843578 | 1688393 | 91.582 | 0.763 | 400723 | 1713748 | 92.96 | 0.77 | 401016 |
| FI18_1_35_205 | m | WY | 35 | F1 | 2985074 | 2906901 | 97.381 | 0.864 | 396332 | 2944441 | 98.64 | 0.87 | 393787 |
| FI18_1_35_23 | m | WY | 35 | F1 | 2660236 | 2517194 | 94.623 | 0.845 | 390549 | 2550049 | 95.86 | 0.85 | 388521 |
| FI18_1_35_26 | m | WY | 35 | F1 | 3518410 | 3419253 | 97.182 | 0.848 | 521155 | 3465568 | 98.50 | 0.85 | 519575 |
| FI18_1_35_27 | m | YY | 35 | F1 | 4180740 | 4018770 | 96.126 | 0.859 | 566600 | 4073081 | 97.42 | 0.86 | 564290 |
| FI18_1_35_30 | m | YY | 35 | F1 | 3154926 | 3052328 | 96.748 | 0.883 | 356336 | 3091011 | 97.97 | 0.89 | 351995 |
| FI18_1_35_34 | m | YY | 35 | F1 | 3944846 | 3793771 | 96.170 | 0.857 | 540650 | 3844661 | 97.46 | 0.86 | 539733 |
| FI18_1_35_38 | m | YY | 35 | F1 | 2862154 | 2762897 | 96.532 | 0.861 | 383162 | 2799925 | 97.83 | 0.86 | 380537 |
| FI18_1_35_4 | m | WY | 35 | F1 | 1577512 | 1536243 | 97.384 | 0.808 | 295506 | 1555941 | 98.63 | 0.87 | 438536 |
| FI18_1_35_41 | m | YY | 35 | F1 | 3559782 | 3367393 | 94.595 | 0.869 | 441184 | 3410928 | 95.82 | 0.81 | 293362 |
| FI18_1_35_42 | m | YY | 35 | F1 | 2472706 | 2299419 | 92.992 | 0.846 | 353007 | 2327801 | 94.14 | 0.85 | 351126 |
| FI18_1_35_44 | m | WY | 35 | F1 | 2710278 | 2619093 | 96.636 | 0.842 | 414429 | 2655136 | 97.97 | 0.84 | 411613 |
| FI18_1_35_46 | m | YY | 35 | F1 | 4262616 | 4140732 | 97.141 | 0.862 | 570989 | 4198044 | 98.49 | 0.86 | 570002 |
| FI18_1_35_48 | m | WY | 35 | F1 | 3216496 | 2916095 | 90.661 | 0.856 | 421370 | 2953800 | 91.83 | 0.86 | 418047 |
| FI18_1_35_50 | m | WY | 35 | F1 | 2489832 | 2423228 | 97.325 | 0.848 | 368727 | 2455548 | 98.62 | 0.85 | 366764 |
| FI18_1_35_55 | m | WY | 35 | F1 | 2359414 | 2275714 | 96.453 | 0.820 | 408809 | 2306363 | 97.75 | 0.82 | 407863 |
| FI18_1_35_63 | m | WY | 35 | F1 | 2052154 | 1974678 | 96.225 | 0.825 | 344993 | 2000431 | 97.48 | 0.83 | 343929 |
| FI18_1_35_64 | m | WY | 35 | F1 | 1527192 | 1485772 | 97.288 | 0.782 | 323976 | 1505656 | 98.59 | 0.79 | 323437 |
| FI18_1_35_7 | m | YY | 35 | F1 | 5425568 | 5283782 | 97.387 | 0.888 | 593717 | 5352263 | 98.65 | 0.89 | 588934 |
| FI18_1_35_71 | m | WY | 35 | F1 | 2400100 | 2329217 | 97.047 | 0.819 | 421606 | 2360482 | 98.35 | 0.82 | 420247 |
| FI18_1_35_75 | m | WY | 35 | F1 | 964512 | 751542 | 77.919 | 0.778 | 166550 | 758586 | 78.65 | 0.78 | 165028 |
| FI18_1_35_77 | m | YY | 35 | F1 | 3730104 | 3620632 | 97.065 | 0.865 | 488724 | 3666177 | 98.29 | 0.87 | 488414 |
| FI18_1_35_8 | m | YY | 35 | F1 | 3452032 | 3358858 | 97.301 | 0.846 | 518108 | 3403069 | 98.58 | 0.85 | 515878 |
| FI18_1_35_80 | m | WY | 35 | F1 | 2593944 | 2496331 | 96.237 | 0.818 | 454804 | 2533709 | 97.68 | 0.82 | 453525 |
| FI18_1_35_87 | m | YY | 35 | F1 | 1765316 | 1719243 | 97.390 | 0.820 | 308853 | 1741749 | 98.66 | 0.82 | 307339 |
| FI18_1_35_88 | m | YY | 35 | F1 | 4767910 | 4573602 | 95.925 | 0.866 | 611810 | 4636650 | 97.25 | 0.87 | 607947 |
| FI18_1_35_90 | m | YY | 35 | F1 | 3354264 | 3252455 | 96.965 | 0.878 | 395524 | 3293166 | 98.18 | 0.88 | 392171 |
| FI18_1_35_95 | m | YY | 35 | F1 | 4430800 | 4302387 | 97.102 | 0.842 | 678160 | 4362196 | 98.45 | 0.84 | 678885 |
| FI18_1_35_96 | m | WY | 35 | F1 | 2715398 | 2640153 | 97.229 | 0.835 | 436057 | 2676128 | 98.55 | 0.84 | 435103 |
| FI18_1_35_97 | m | WY | 35 | F1 | 4886316 | 4653904 | 95.244 | 0.899 | 471477 | 4712210 | 96.44 | 0.90 | 467161 |
| FI18_1_29_14_1 | excluded due to low mapping | | 29 | F1 | 5754900 | 274244 | 4.765 |  |  | 278459 | 4.84 |  |  |
| FI18_1_29_20_1 | excluded due to low mapping | | 29 | F1 | 691934 | 153766 | 22.223 |  |  | 155963 | 22.54 |  |  |
| FI18_1_29_3_1 | excluded due to low mapping | | 29 | F1 | 6673428 | 349921 | 5.243 |  |  | 352906 | 5.29 |  |  |
| FI18_1_29_59_1 | excluded due to low mapping | | 29 | F1 | 2940246 | 206153 | 7.011 |  |  | 208804 | 7.10 |  |  |
| FI18_1_29_97_1 | excluded due to low mapping | | 29 | F1 | 7207076 | 1488963 | 20.660 |  |  | 1508887 | 20.94 |  |  |
| FI18_1_30_96_1 | excluded due to low mapping | | 30 | F1 | 1115724 |  |  |  |  |  |  |  |  |
| FI18_1_32_50_2_1 | excluded due to low mapping | | 32 | F1 | 9689116 | 368989 | 3.808 |  |  | 373251 | 3.85 |  |  |
| FI18_1_32_74_1 | excluded due to low mapping | | 32 | F1 | 6430210 | 1215692 | 18.906 |  |  | 1229932 | 19.13 |  |  |
| FI18_1_32_94_1 | excluded due to low mapping | | 32 | F1 | 6933950 | 5839 | 0.084 |  |  | 5963 | 0.09 |  |  |
| FI18_1_35_53_1 | excluded due to low mapping | | 35 | F1 | 11105008 | 10313 | 0.093 |  |  | 10423 | 0.09 |  |  |
| FI18_1_35_91_1 | excluded due to low mapping | | 35 | F1 | 9853700 | 62965 | 0.639 |  |  | 63712 | 0.65 |  |  |
| FI18_1_35_94_1 | excluded due to low mapping | | 35 | F1 | 6052848 | 1562498 | 25.814 |  |  | 1582649 | 26.15 |  |  |

Supplementary file 1F: Sample list of all wild samples used.

| ID | Locality | Latitude | Longitude | Region | Collection year | Sex | Colour | Used in GWAS | Total sequenced reads | YY Mapped reads | YY % mapped reads | YY duplication | YY Reads retained after deduplication | YY average read depth | WW mapped read | WW % mapped | WW duplication | WW Reads retained after deduplication | WW average read depth |
| --- | --- | --- | --- | --- | --- | --- | --- | --- | --- | --- | --- | --- | --- | --- | --- | --- | --- | --- | --- |
| CAM015132 | Huosiaisnotko, Laukaa | 62.38634 | 25.81796 | Central Finland | 2018 | Male | Y | x | 99337203 | 95728163 | 96.37 | 0.13 | 83260117 | 17.382 | 99278718 | 99.02 | 0.14 | 98307916 | 17.6129 |
| CAM015133 | Huosiaisnotko, Laukaa | 62.38634 | 25.81796 | Central Finland | 2018 | Male | Y | x | 91549356 | 88141528 | 96.28 | 0.14 | 75592171 | 15.8571 | 91510105 | 98.94 | 0.15 | 90540109 | 16.0169 |
| CAM015134 | Haralanharju, Kangasala | 61.53456355 | 24.08097897 | Central Finland | 2018 | Male | Y | x | 84051672 | 81125511 | 96.52 | 0.14 | 69937864 | 14.6407 | 84005409 | 99.03 | 0.14 | 83188228 | 14.8749 |
| CAM015135 | Heposuo, Laukaa | 62.34784 | 25.82785 | Central Finland | 2018 | Male | Y | x | 82188518 | 79203877 | 96.37 | 0.14 | 68088328 | 14.2128 | 82119542 | 99 | 0.15 | 81299902 | 14.3792 |
| CAM015136 | Huosiaisnotko, Laukaa | 62.38634 | 25.81796 | Central Finland | 2018 | Male | Y | x | 83831177 | 80915884 | 96.52 | 0.14 | 69802421 | 14.6376 | 83766436 | 99.03 | 0.14 | 82952260 | 14.842 |
| CAM015137 | Mäyrämäki, Jyväskylä | 62.22814 | 25.64794 | Central Finland | 2018 | Male | W | x | 90325260 | 86804526 | 96.10 | 0.14 | 74784341 | 15.8328 | 90270209 | 98.99 | 0.14 | 89355999 | 16.0069 |
| CAM015138 | Heposuo, Laukaa | 62.34784 | 25.82785 | Central Finland | 2018 | Male | W | x | 83258916 | 80277374 | 96.42 | 0.14 | 69295411 | 14.4791 | 83204225 | 98.98 | 0.14 | 82355152 | 14.675 |
| CAM015139 | Lautaperä, Keuruu | 62.18709 | 24.87121 | Central Finland | 2018 | Male | W | x | 91929373 | 88159990 | 95.90 | 0.14 | 75864499 | 15.7166 | 91852724 | 99.04 | 0.15 | 90968838 | 15.9305 |
| CAM015140 | Heposuo, Laukaa | 62.34784 | 25.82785 | Central Finland | 2018 | Male | W | x | 85888608 | 82609774 | 96.18 | 0.14 | 71419687 | 14.8998 | 85825617 | 98.96 | 0.14 | 84937070 | 15.1099 |
| CAM015141 | Huosiaisnotko, Laukaa | 62.38634 | 25.81796 | Central Finland | 2018 | Male | W | x | 76588254 | 73780022 | 96.33 | 0.14 | 63477797 | 13.3699 | 76558659 | 99.01 | 0.15 | 75803292 | 13.5154 |
| CAM015192 | Heposuo, Laukaa | 62.34784 | 25.82785 | Central Finland | 2018 | Male | Y | x | 80404515 | 77653009 | 96.58 | 0.15 | 65662866 | 13.7736 | 80351585 | 98.85 | 0.16 | 79428391 | 14.0112 |
| CAM015193 | Heposuo, Laukaa | 62.34784 | 25.82785 | Central Finland | 2018 | Male | Y | x | 58498076 | 56522266 | 96.62 | 0.13 | 49442117 | 10.4086 | 58462162 | 99.04 | 0.13 | 57903161 | 10.5802 |
| CAM015194 | Heposuo, Laukaa | 62.34784 | 25.82785 | Central Finland | 2018 | Male | Y | x | 65622607 | 63282509 | 96.43 | 0.14 | 54683278 | 11.5851 | 65581436 | 98.98 | 0.14 | 64914704 | 11.7449 |
| CAM015195 | Mäyrämäki, Jyväskylä | 62.22814 | 25.64794 | Central Finland | 2018 | Male | Y | x | 58154455 | 56272290 | 96.76 | 0.12 | 49706191 | 10.5543 | 58119500 | 99.02 | 0.12 | 57547059 | 10.6945 |
| CAM015196 | Mäyrämäki, Jyväskylä | 62.22814 | 25.64794 | Central Finland | 2018 | Male | Y | x | 83371214 | 80690761 | 96.78 | 0.14 | 69714679 | 14.7052 | 83320131 | 99.05 | 0.14 | 82530724 | 14.8955 |
| CAM015197 | Mäyrämäki, Jyväskylä | 62.22814 | 25.64794 | Central Finland | 2018 | Male | W | x | 68675694 | 66496196 | 96.83 | 0.13 | 58102548 | 12.2659 | 68633799 | 99.08 | 0.13 | 68004964 | 12.4399 |
| CAM015198 | Mäyrämäki, Jyväskylä | 62.22814 | 25.64794 | Central Finland | 2018 | Male | W | x | 75453002 | 72010183 | 95.44 | 0.24 | 55086736 | 11.385 | 75405484 | 98.87 | 0.25 | 74551498 | 11.5827 |
| CAM015199 | Mäyrämäki, Jyväskylä | 62.22814 | 25.64794 | Central Finland | 2018 | Male | W | x | 69939593 | 67389470 | 96.35 | 0.14 | 58195017 | 12.5471 | 69889281 | 99.04 | 0.14 | 69214869 | 12.7278 |
| CAM015200 | Mäyrämäki, Jyväskylä | 62.22814 | 25.64794 | Central Finland | 2018 | Male | W | x | 55198189 | 53350748 | 96.65 | 0.12 | 47208830 | 10.2012 | 56405976 | 99.08 | 0.12 | 54666270 | 10.3219 |
| CAM015201 | Mäyrämäki, Jyväskylä | 62.22814 | 25.64794 | Central Finland | 2018 | Male | W | x | 92938532 | 88633493 | 95.37 | 0.24 | 67242769 | 13.8414 | 92892355 | 98.88 | 0.25 | 91856037 | 14.0357 |
| CAM015142 | Voiaskintie, Ulrikasund | 60.43466 | 25.32835 | Southern Finland | 2018 | Male | Y | x | 84316506 | 80894323 | 95.94 | 0.13 | 70140012 | 14.8534 | 84287184 | 99.04 | 0.14 | 83480957 | 15.0015 |
| CAM015143 | Tvärminne, Hanko | 59.846 | 23.185 | Southern Finland | 2018 | Male | Y | x | 81341932 | 78370587 | 96.35 | 0.14 | 67261188 | 14.2685 | 81283183 | 99 | 0.15 | 80472992 | 14.4447 |
| CAM015144 | Tvärminne, Hanko | 59.846 | 23.185 | Southern Finland | 2018 | Male | Y | x | 88405065 | 85421712 | 96.63 | 0.14 | 73193505 | 15.1229 | 88352550 | 98.97 | 0.15 | 87440926 | 15.3091 |
| CAM015145 | Tvärminne, Hanko | 59.846 | 23.185 | Southern Finland | 2018 | Male | Y | x | 80894075 | 77989309 | 96.41 | 0.13 | 67679132 | 14.0029 | 80841879 | 98.97 | 0.14 | 80008747 | 14.195 |
| CAM015146 | Tvärminne, Hanko | 59.846 | 23.185 | Southern Finland | 2018 | Male | Y | x | 81136931 | 78241764 | 96.43 | 0.13 | 67854265 | 14.2953 | 81086172 | 98.89 | 0.14 | 80187814 | 14.451 |
| CAM015147 | Voiaskintie, Ulrikasund | 60.43466 | 25.32835 | Southern Finland | 2018 | Male | W | x | 75094177 | 72333859 | 96.32 | 0.13 | 62642058 | 13.4071 | 75042444 | 99 | 0.14 | 74295482 | 13.5672 |
| CAM015148 | Voiaskintie, Ulrikasund | 60.43466 | 25.32835 | Southern Finland | 2018 | Male | W | x | 89834991 | 86506811 | 96.30 | 0.14 | 74333381 | 15.7749 | 89777636 | 99 | 0.15 | 88882799 | 15.9597 |
| CAM015149 | Mosabackantie, Sipoo | 60.36973 | 24.54084 | Southern Finland | 2018 | Male | W | x | 73648890 | 71065467 | 96.49 | 0.13 | 61618406 | 13.0722 | 73604268 | 99.03 | 0.14 | 72886794 | 13.2296 |
| CAM015150 | Voiaskintie, Ulrikasund | 60.43466 | 25.32835 | Southern Finland | 2018 | Male | W | x | 72378681 | 69440673 | 95.94 | 0.13 | 60321260 | 12.834 | 72332606 | 98.98 | 0.14 | 71594111 | 13.0016 |
| CAM015151 | Voiaskintie, Ulrikasund | 60.43466 | 25.32835 | Southern Finland | 2018 | Male | W | x | 80232467 | 76779318 | 95.70 | 0.14 | 66143339 | 13.6987 | 80159712 | 99.06 | 0.15 | 79405646 | 13.9165 |
| CAM015154 | Kanaküla 2 | 58.26328 | 25.11927 | Estonia | 2018 | Male | W | x | 71789797 | 69217313 | 96.42 | 0.13 | 60293429 | 12.7039 | 71746939 | 98.93 | 0.13 | 70982660 | 12.8702 |
| CAM015155 | Kanaküla 1.2 | 58.15794 | 25.8694 | Estonia | 2018 | Male | W | x | 90309170 | 87203546 | 96.56 | 0.14 | 75335592 | 15.3226 | 90247219 | 98.99 | 0.14 | 89333141 | 15.5947 |
| CAM015158 | Kanaküla 4 | 58.274533 | 25.117817 | Estonia | 2018 | Male | W | x | 77232567 | 74330844 | 96.24 | 0.14 | 64205532 | 13.123 | 82512306 | 98.99 | 0.14 | 76392613 | 13.3823 |
| CAM015159 | Kanaküla 2 | 58.26328 | 25.11927 | Estonia | 2018 | Male | W | x | 79351392 | 76638691 | 96.58 | 0.13 | 66913224 | 13.7015 | 77179045 | 98.98 | 0.13 | 78483602 | 13.8975 |
| CAM015162 | Thieves Hill, Aultmore, Keith | 57.575222 | -3.048778 | Scotland | 2015 | Male | Y | x | 82938651 | 79794024 | 96.21 | 0.15 | 67813099 | 14.2972 | 79287964 | 98.99 | 0.16 | 82016446 | 14.5358 |
| CAM015163 | Portknockie, Buckie | 57.702785 | -2.879435 | Scotland | 2015 | Male | Y | x | 83269393 | 80197317 | 96.31 | 0.14 | 68632488 | 14.4383 | 82886426 | 98.95 | 0.15 | 82338256 | 14.6507 |
| CAM015165 | Portknockie, Buckie | 57.702785 | -2.879435 | Scotland | 2015 | Male | Y | x | 71071794 | 47490 | 0.07 | 0.14 | 40949 | 0.00243509 | removed due to low mapping % | | |  |  |
| CAM015170 | Findlater Castle, Portsoy | 57.69157 | -2.77265 | Scotland | 2015 | Male | Y | x | 59557227 | 57359570 | 96.31 | 0.13 | 50112976 | 10.4116 | 71072090 | 0.79 | 0.13 | 58867297 | 10.5789 |
| CAM015202 | F1 offspring of wild parents | NA | NA | Scotland | 2015 | Male | Y | x | 59937755 | 58011585 | 96.79 | 0.13 | 50720420 | 10.7925 | 59903009 | 99 | 0.13 | 59301604 | 10.9623 |
| CAM015203 | F1 offspring of wild parents | NA | NA | Scotland | 2015 | Male | Y | x | 55068668 | 53331495 | 96.85 | 0.12 | 46913748 | 9.92293 | 57251863 | 99.03 | 0.13 | 54495189 | 10.0522 |
| CAM015204 | F1 offspring of wild parents | NA | NA | Scotland | 2015 | Male | Y | x | 64822930 | 62348914 | 96.18 | 0.13 | 54148683 | 11.475 | 64780636 | 98.08 | 0.14 | 63535554 | 11.6182 |
| CAM015206 | F1 offspring of wild parents | NA | NA | Scotland | 2015 | Male | Y | x | 87767164 | 84630216 | 96.43 | 0.16 | 70781662 | 14.6903 | 87719259 | 98.9 | 0.17 | 86758116 | 14.9508 |
| CAM015207 | F1 offspring of wild parents | NA | NA | Scotland | 2015 | Male | Y | x | 62230737 | 58239265 | 93.59 | 0.12 | 51177246 | 10.9483 | 62197559 | 96.13 | 0.13 | 59791385 | 11.1071 |
| CAM015208 | F1 offspring of wild parents | NA | NA | Scotland | 2015 | Male | Y | x | 47567413 | 46042450 | 96.79 | 0.12 | 40522208 | 8.60095 | 64848486 | 98.96 | 0.12 | 64176574 | 11.8856 |
| CAM015209 | F1 offspring of wild parents | NA | NA | Scotland | 2015 | Male | Y | x | 119873564 | 114836831 | 95.80 | 0.17 | 95610964 | 19.8179 | 119796501 | 98.93 | 0.18 | 118513971 | 20.175 |
| CAM015211 | F1 offspring of wild parents | NA | NA | Scotland | 2015 | Male | Y | x | 47171055 | 45865834 | 97.23 | 0.11 | 40747916 | 8.70432 | 63799250 | 99.04 | 0.11 | 63185074 | 11.9608 |
| CAM15072 | Tvärminne, Hanko | 59.846 | 23.185 | Southern Finland |  | Male | Y |  | These samples were added to later analyses using the WW genome | | | | |  |  |  | 0.08 | 71280369 | 14.1515 |
| CAM015073 | Tvärminne, Hanko | 59.846 | 23.185 | Southern Finland |  | Male | Y |  |  |  |  |  |  |  | 68638162 | 99.02 | 0.08 | 67967326 | 13.4971 |
| CAM015078 | Tvärminne, Hanko | 59.846 | 23.185 | Southern Finland |  | Male | W |  |  |  |  |  |  |  | 70063666 | 99.04 | 0.08 | 69389996 | 13.7484 |
| CAM15084 | Tvärminne, Hanko | 59.846 | 23.185 | Southern Finland |  | Male | W |  |  |  |  |  |  |  |  |  | 0.08 | 70835562 | 13.9317 |
| CAM015085 | Tvärminne, Hanko | 59.846 | 23.185 | Southern Finland |  | Male | W |  |  |  |  |  |  |  | 49250064 | 99.05 | 0.09 | 48782086 | 9.66459 |
| CAM015086 | Tvärminne, Hanko | 59.846 | 23.185 | Southern Finland |  | Male | Y |  |  |  |  |  |  |  | 57402371 | 98.98 | 0.08 | 56815328 | 11.2504 |
| CAM015088 | Tvärminne, Hanko | 59.846 | 23.185 | Southern Finland |  | Male | Y |  |  |  |  |  |  |  | 65556920 | 99.02 | 0.09 | 64913551 | 12.7004 |
| CAM015089 | Tvärminne, Hanko | 59.846 | 23.185 | Southern Finland |  | Male | W |  |  |  |  |  |  |  | 67913274 | 99.02 | 0.09 | 67245909 | 13.2502 |
| CAM015091 | Tvärminne, Hanko | 59.846 | 23.185 | Southern Finland |  | Male | W |  |  |  |  |  |  |  | 66279795 | 99.04 | 0.09 | 65642031 | 12.9068 |
| CAM015094 | Tvärminne, Hanko | 59.846 | 23.185 | Southern Finland |  | Male | Y |  |  |  |  |  |  |  | 71591177 | 98.88 | 0.10 | 70791957 | 13.6097 |

Supplementary file 1G: CRISPR sgRNAs sequences.

| **sgRNA name** | **Target sequence** | **Starting location in *valkea*** | **PAM** | **Specificity score %** | **Activity score** |
| --- | --- | --- | --- | --- | --- |
| Val1A | CTGAGAACACGGTACCTACG | WW_tarseq_419_arrow:7,014,923 | GGG | 62.5 | 0.68 |
| Val2A | ACACCCACAGTCTATTGCAG | WW_tarseq_419_arrow:7,021,659 | TGG | 62.5 | 0.505 |
| Val2B | TCGTGGCATTCTGCCGGCCG | WW_tarseq_419_arrow:7,021,588 | CGG | 62.5 | 0.698 |
| Val3A | CTCGGGGGAAGTATACACTT | WW_tarseq_419_arrow:7,031,836 | CGG | 83.3 | 0.722 |
| Val3B | CTTCGCTTACATCAGTGACA | WW_tarseq_419_arrow:7,031,950 | CGG | 83.3 | 0.754 |

Supplementary file 1H: Elution gradient used in pheomelanin HPLC analysis.

| **Time (s)** | **%A** | **%B** |
| --- | --- | --- |
| 0 | 96 | 4 |
| 0.2 | 96 | 4 |
| 0.3 | 94 | 6 |
| 20 | 94 | 6 |
| 21 | 60 | 40 |
| 35 | 40 | 60 |
| 39 | 40 | 60 |
| 42 | 96 | 4 |

Supplementary file 1I: Waveform of disposable working electrode in pheomelanin analysis.

| **Time (s)** | **Potential (V)** | **Integration** |
| --- | --- | --- |
| 0 | 0.13 |  |
| 0.04 | 0.13 |  |
| 0.05 | 0.45 |  |
| 0.21 | 0.45 | Begin |
| 0.56 | 0.45 | End |
| 0.57 | -1.67 |  |
| 0.58 | -1.67 |  |
| 0.59 | 0.93 |  |
| 0.6 | 0.13 |  |
