# Supplementary material for: Colour polymorphism associated with a gene duplication in male wood tiger moths
Source: eLife. 2023 Oct 30;12:e80116. doi: 10.7554/eLife.80116 (PMC10635649; doi:10.7554/eLife.80116)

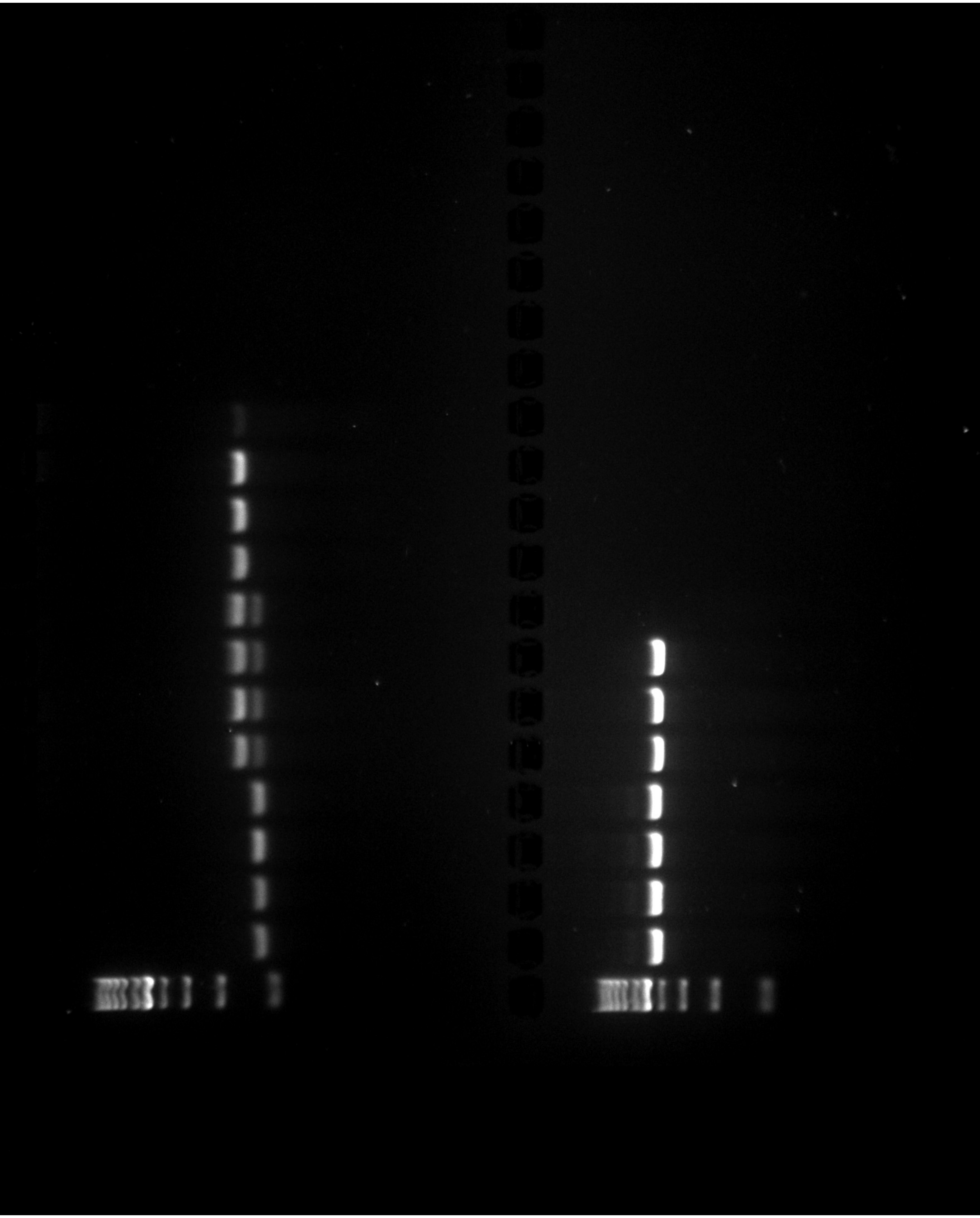

Supplement: Figure 2—figure supplement 4—source data 1. [file elife-80116-fig2-figsupp4-data1.pdf]
